# The Role of WNT3A Protein and Gene Variants in Allergic Rhinitis: A Case-Control Study

**DOI:** 10.3390/cimb46090565

**Published:** 2024-08-29

**Authors:** Durkadin Demir Eksi, Huseyin Gunizi

**Affiliations:** 1 Department of Medical Biology, School of Medicine, Alanya Alaaddin Keykubat University, Antalya 07425, Türkiye; 2 Department of Otolaryngology, Antalya City Hospital, Antalya 07080, Türkiye; drgunizi@gmail.com

**Keywords:** allergic rhinitis, WNT3A, polymorphisms, biomarkers, genetics, respiratory allergies

## Abstract

Allergic rhinitis (AR) is a prevalent inflammatory disorder of the upper respiratory tract, driven by allergen exposure. Understanding mechanisms and identifying biomarkers for AR could significantly impact diagnosis and treatment. This study aimed to investigate the association between serum Wingless-Type MMTV Integration Site Family, Member 3A (WNT3A) protein levels, *WNT3A* polymorphisms, and AR. A cohort of 92 AR patients and 86 healthy controls was recruited. Serum WNT3A levels were measured by enzyme-linked immunosorbent assay (ELISA). *WNT3A* gene polymorphisms (rs752107 and rs3121310) were analyzed using Polymerase Chain Reaction- Restriction Fragment Length Polymorphism (PCR-RFLP) method. The study revealed significantly higher serum WNT3A levels in AR patients compared to controls (*p* < 0.0001). The impact of WNT3A in the differential diagnosis of AR was determined to be moderate, with an area under the curve (AUC) value of 0.67 (95% Confidence Interval: 0.59–0.75) based on the receiver operating characteristic (ROC) curve analysis. The rs3121310 polymorphism showed a significant association with the GA genotype more prevalent in controls (*p* < 0.05). However, no significant relationship was observed between rs3121310 genotypes and clinical parameters of the patients. These findings suggest a role for WNT3A in AR pathogenesis, given the elevated serum levels in patients. Larger cohort studies are needed to validate these findings and explore serum WNT3A levels as a biomarker for AR diagnosis and treatment monitoring.

## 1. Introduction

Allergic rhinitis (AR) is an upper respiratory tract disease characterized by nasal congestion, rhinorrhea, sneezing, and nasal itching symptoms caused by inhaled allergens, followed by mucosal inflammation [1]. AR is a chronic inflammatory disease mediated by Immunoglobulin E (IgE) that negatively impacts patients’ quality of life and is the most common clinical manifestation among respiratory allergies [2]. As a prevalent allergic inflammatory rhinopathy, AR is affecting approximately 20–30% of adults and 40% of children worldwide [2,3]. The classification of AR has undergone revision by the Allergic Rhinitis and its Impact on Asthma (ARIA) group recently. This updated classification integrates an evaluation of symptom frequency and duration. Intermittent AR (IAR) is defined as the presence of symptoms for fewer than four days per week or lasting for less than four consecutive weeks. Persistent AR (PER) is characterized by symptoms persisting for more than four days per week and lasting for more than four consecutive weeks. Furthermore, the revised classification also incorporates a severity scale ranging from mild to moderate-severe [4].

The Wingless-Type MMTV Integration Site Family (Wnt) signaling pathway is known to play roles in cell proliferation, cell differentiation, embryonic development, and immune system regulation. Extracellular signals are transmitted by ligands such as WNT, Member 1 (WNT1), WNT, Member 3A (WNT3A), and WNT, Member 5A (WNT5A) [5]. Studies have indicated the regulatory role of the Wnt signaling pathway in modulating inflammatory responses in airway diseases like asthma [6]. WNT3A has been shown to activate the WNT/β-catenin signaling pathway in mature human mast cells. This activation leads to an increase in Interleukin 8 (*IL-8*) and C-C Motif Chemokine Ligand 8 (*CCL8*) mRNA expression and the release of IL-8 protein. Consequently, researchers have proposed that the activation of mast cells by WNT3A may be effective in stimulating immune cells in diseases where *WNT3A* expression is increased, such as asthma [7]. Li et al. demonstrated that inhibition of the Wnt/β-catenin pathway in an Ovalbumin (OVA)-induced mouse AR model led to a reduction in eosinophil and mast cell numbers. Moreover, serum levels of IgE, Interferon-gamma (INF-γ), IL-1β, IL-4, IL-17, β-catenin, and Glycogen synthase kinase-3 beta (GSK-3β) were downregulated. These findings highlight the potential of targeting the Wnt/β-catenin pathway as a novel therapeutic approach for the treatment of AR [8].

Atopic diseases, including allergic rhinoconjunctivitis, asthma, atopic dermatitis, and food allergies, are generally familial and associated with numerous genetic loci [9]. Therefore, we aimed to investigate the relationship between serum WNT3A protein levels and *WNT3A* polymorphisms in patients diagnosed with AR in this study.

## 2. Materials and Methods

### 2.1. Study Population

The study population comprised 92 adult patients who were diagnosed with AR and sought treatment at the Ear, Nose, and Throat (ENT) Diseases outpatient clinic of Alanya Alaaddin Keykubat University Alanya Training and Research Hospital. Additionally, 86 healthy adult individuals within the same age range were recruited as controls. Ethical approval for the study was obtained from the Alanya Alaaddin Keykubat University Clinical Research Ethics Committee (decision number: 05/01, dated 10 March 2021), and informed consent was obtained from all participants. The patient group consisted of Turkish individuals diagnosed solely with AR, without any concurrent medical conditions. Exclusion criteria included patients with a history of malignancy, pregnant women, individuals with a history of steroid use for any medical condition, obesity, diabetes, signs of metabolic syndrome, or those who smoke. The control group comprised healthy Turkish individuals who had not previously received treatment for AR, had not undergone any malignancy treatment, and did not smoke. Clinical data, including biochemical parameters such as total IgE levels, and clinical characteristics such as duration of diagnosis, frequency of symptoms, and disease severity (classified as mild, moderate, or severe), were collected for each patient.

### 2.2. Determination of Serum WNT3A Protein Levels by ELISA

Blood samples of 5 mL each were collected into gel tubes from 92 patients and 86 healthy control individuals and allowed to clot at room temperature. After clotting, the samples were centrifuged at 2000 rpm for 10 min. Following centrifugation, the serum samples were aliquoted into 1 mL Eppendorf tubes and stored at −20 °C until the day of analysis. On the day of analysis, serum samples from both patient and control groups were thawed at room temperature. Enzyme-linked immunosorbent assay (ELISA) analysis was performed according to the manufacturer’s instructions using the Human Protein WNT3A ELISA kit (BT Lab, Jiaxing, China). Briefly, standards and samples were run in triplicate. The required number of strips was determined and inserted into the frame. For the assay, 50 µL of the standard was added to the designated standard wells. Next, 40 µL of the sample was added to the sample wells, followed by the addition of 10 µL of Human WNT3A antibody to each sample well. Subsequently, 50 µL of streptavidin-HRP was added to both the sample and standard wells (excluding the blank control well). The plate was gently mixed, covered with a sealer, and incubated for 60 min at 37 °C. After incubation, the sealer was removed, and the plate was washed five times with washing buffer by using an automated washing machine (Biotek Synergy Microplate Washer, BioTek Instruments, Inc., Santa Clara, CA, USA). Next, 50 µL of substrate solution A was added to each well, followed by 50 µL of substrate solution B. The plate was covered with a new sealer and incubated in the dark for 10 min at 37 °C. After incubation, 50 µL of Stop Solution was added to each well. The optical density (OD) of each well at 450 nm was determined immediately using a microplate reader (Biotek Synergy H1 Multimode Reader, BioTek Instruments, Inc., Santa Clara, CA, USA).

### 2.3. Genomic DNA Extraction and Genoytping

Blood samples were collected in K3 EDTA tubes from both patients and healthy controls for genomic DNA (gDNA) extraction. The Roche High Pure PCR Template Preparation kit Version 20 (Roche Diagnostics GmbH, Mannheim, Germany) was utilized for gDNA extraction. Extracted gDNA samples were stored at −20 °C until further analysis. The determination of the rs752107 (C>T) and rs3121310 (G>A) polymorphisms located in the *WNT3A* gene (NM_033131.4) in patient and control individuals was performed using the Polymerase Chain Reaction-Restriction Fragment Length Polymorphism (PCR-RFLP) method. The AluI restriction enzyme (New England Biolabs Ltd., Ipswich, MA, USA) was used for genotyping the rs752107 SNP, and the HpaII restriction enzyme (New England Biolabs Ltd., Ipswich, MA, USA) was used for rs3121310 SNP, following the manufacturer’s instructions. Following digestion, cleavage products were separated on a 3.0% (*w*/*v*) agarose gel via electrophoresis and visualized using the G:BOX Chemi XRQ gel documentation system (Syngene, Cambridge, UK). Thermo Scientific GeneRuler 50 bp DNA Ladder (Thermo Fisher Scientific, Waltham, MA, USA) is used as marker. Oligonucleotide primer sequences, annealing temperatures, and PCR-RFLP product sizes are provided in Table 1. For PCR-RFLP data confirmation, 12 DNA samples with different genotypes of the relevant gene regions were randomly selected for Sanger sequencing. Sanger sequencing was performed using the Applied Biosystems 3130 Genetic Analyzer (Applied Biosystems, Foster City, CA, USA).

To assess the association between genetic variants and gene expression profiles, expression quantitative trait loci (eQTL) were analyzed using the GTEx portal database (http://www.gtexportal.org/home/ (accessed on 22 August 2024)), which provides gene expression data across various tissues.

### 2.4. Statistical Analysis

Statistical analyses were performed using GraphPad Prism software version 7.0 (GraphPad Software, San Diego, CA, USA). Discrete variable frequencies between patients and controls were compared using the Chi-square test (Fisher’s exact test), while continuous variable frequencies were assessed using Student’s *t*-test or the Mann–Whitney test for non-parametric variables. Allelic differences were evaluated through 2 × 2 contingency tables with a two-sided Fisher exact test. The odds ratio (OR) was used to evaluate the strength of association, and 95% confidence intervals (CI) were computed. The diagnostic efficacy of WNT3A protein in distinguishing between controls and AR patients was evaluated using the area under the curve (AUC) of the receiver operating characteristic (ROC) curves. An optimal cut-off point was determined by the Youden index. Genetic findings and WNT3A protein levels were compared using the Kruskal–Wallis test. The Hardy–Weinberg equilibrium (HWE) was assessed using an online computational tool (https://www.cog-genomics.org/software/stats (accessed on 24 May 2024)). A significance level of *p* < 0.05 was applied.

## 3. Results

The study included AR patients and healthy controls matched for age and sex. Table 2 presents the demographic characteristics and clinical profiles of the participants in the study.

### 3.1. Serum WNT3A Levels

**Figure 1:**
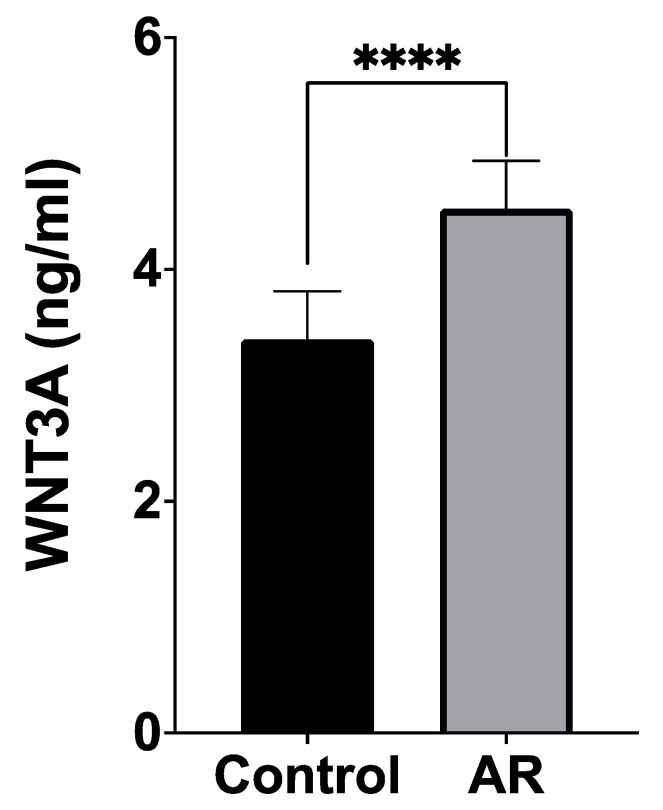
Comparison of serum WNT3A levels between the patient and control groups. (**** *p* < 0.0001).

The serum WNT3A levels of patients were found to be significantly higher compared to the control group (4.369 ± 3.763 ng/mL versus 3.255 ± 3.727 ng/mL, respectively; *p* < 0.0001) (Figure 1).

**Figure 2:**
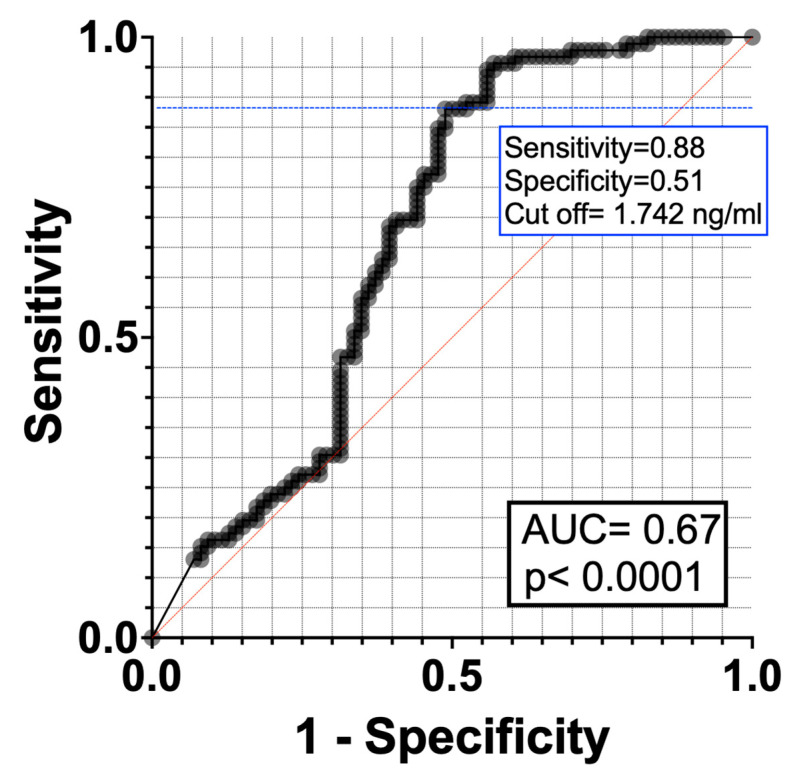
ROC curve of WNT3A protein between control and AR groups. Blue dashed line indicates cut off point. AUC; area under the curve.

The AUC-ROC curve value for WNT3A in discriminating AR was 0.67, with a 95% CI ranging from 0.59 to 0.75 The optimal cut-off point based on the Youden index was 1.742 ng/mL with a sensitivity of 0.88 and a specificity of 0.51 (Figure 2).

On the other hand, there were no significant differences in the serum WNT3A levels among the patients classified by disease characteristics (Table 3).

### 3.2. Distribution of Genotypes

**Figure 3:**
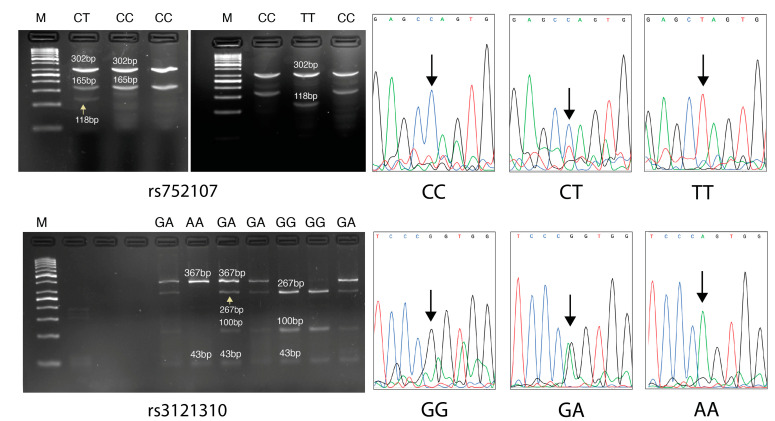
Selected PCR-RFLP gels for both polymorphisms along with their corresponding DNA sequencing electropherogram results. (M: Marker, CC: wild-type, CT: heterozygous mutant, TT: homozygous mutant genotypes for the rs752107 polymorphism. GG: wild-type, GA: heterozygous mutant, AA: homozygous mutant for the rs3121310 polymorphism. The 47 bp product for genotyping the rs752107 polymorphism is not visible on gels likely due to its small quantity; however, this does not affect the accuracy of genotyping. The sizes of PCR-RFLP products are indicated above the products in base pairs (bp), except for those indicated by yellow arrows. The black arrows in the electropherogram images indicate the SNP points in the relevant gene region).

The gel images and electropherograms obtained from Sanger sequencing for the rs752107 and rs3121310 SNPs are shown in Figure 3. The distribution of genotypic findings, allele frequencies, and statistical analysis results for both patient and control groups are presented in Table 4. There was no significant difference in the genotypic distribution of the rs752107 polymorphism between the patient and control groups. However, for the rs3121310 polymorphism, the GA genotype was found to be significantly higher in the control group (*p* = 0.0433) (Table 4).

According to the HWE analysis, the genotypic frequencies of the rs752107 polymorphism in the patient and control groups significantly deviated from HWE (*p* = 0.0147, *p* = 0.00001, respectively). Consequently, the rs752107 polymorphism was excluded from further analysis. However, the genotypic findings of the rs3121310 polymorphism in the AR and control groups exhibited no deviation from HWE (*p* > 0.05) (Table 5).

No statistically significant relationship was observed when comparing the clinical findings, such as symptom duration and disease severity with rs3121310 genotypes among the patients (*p* > 0.05). Furthermore, no statistically significant difference was observed when comparing the WNT3A protein levels with rs3121310 polymorphism genotype data in patients (*p* > 0.05) (Table 6). No results were found regarding eQTL for the association of rs3121310 polymorphism with WNT3A in the whole blood. In addition, rs3121310 was not associated with *WNT3A* expression in the lung (*p* = 0.71).

## 4. Discussion

AR is a disease whose etiopathogenesis has not yet been fully elucidated. While the contribution of genetic factors in the development of the disease is known, it is observed to be genetically heterogeneous. Considering interpopulation differences, there is a need for more genetic studies to be conducted. The available treatments for specific AR phenotypes are not curative and occasionally ineffective. Therefore, new therapeutic approaches are necessary. The Wnt signaling pathway has important functions in developmental processes. It controls various processes such as cell proliferation and self-renewal of stem and progenitor cells. The Wnt signaling pathway includes Wnt/β-catenin, Wnt/PCP, and Wnt/Ca2+ pathways. β-catenin functions as a key signal transducer in this pathway. Due to the role of the Wnt/β-catenin signaling pathway in embryogenesis and tissue homeostasis, it has been found to be closely associated with the development of various human diseases and cancers. The Wnt/β-catenin signaling pathway is evolutionarily conserved and regulates various biological processes throughout mammalian development and adult life. Additionally, it has both anti-inflammatory and pro-inflammatory functions [8]. Recent studies demonstrate the effects of the Wnt signaling pathway on both acquired and innate immunity. One of the Wnt ligands that regulates the immune system response through immune system cells and airway epithelial and fibroblast cells is WNT3A [5]. The canonical Wnt signaling pathway plays a significant role in the development and differentiation of the airways and alveoli [10,11]. Within the scope of our study, preliminary data have been obtained regarding the relationship between the Wnt signaling pathway and AR.

The Wnt/β-catenin signaling pathway is directly activated by WNT3A. Functional mutations of the *WNT3A* gene may have an impact on the etiology of cardio-cerebrovascular diseases [12]. To date, there has been no study investigating the association between *WNT3A* polymorphisms and AR. Our study is the first study investigating the relationship between circulating WNT3A, *WNT3A* SNPs, and AR in human subjects. The rs752107 polymorphism is located in 3′untranslated region (3′-UTR), which binds to MicroRNAs (miRNAs) to regulate gene expression [13]. The existence of the C allele was expected to create a more robust binding site for has-miR-892b, leading to the suppression of *WNT3A* gene expression [12]. Bone mineral density variation and cleft palate have been linked to the *WNT3A* rs752107 polymorphism [14,15]. In our study, a strong deviation from HWE was observed in the genotypic frequencies of the rs752107 polymorphism particularly within the control group. Following advanced analyses, the rs752107 polymorphism was excluded from further investigation. The genotyping experiments were conducted uniformly within a single laboratory, with personnel blinded to the specimen type (patient vs. control). The deviation from HWE for the rs752107 polymorphism could potentially stem from various genetic factors, recent population admixture, sample size, as well as population stratification specific to that particular SNP [16,17]. SNPs should be screened in larger cohorts for more robust statistical power and comprehensive analysis.

The rs3121310 polymorphism is a variation localized in the intronic region of *WNT3A* gene and associated with male patients affected by non-syndromic cleft lip palate [14]. The association of this polymorphism with respiratory diseases has not been investigated to date. In our study, the GA genotype of the rs3121310 polymorphism was found to be significantly higher in the control group compared to the patient group (*p* = 0.0433 [OR (95% CI): 0.5174 (0.280–0.978)]). Intronic SNPs affect gene expression by influencing alternative splicing, genomic imprinting, the regulation of gene expression through long non-coding RNAs (lncRNAs), chromatin looping, the transcription process, and the formation of premature stop codons [18]. The role of the rs3121310 polymorphism in these processes is still unknown. In the current study, despite the absence of a statistically significant difference, the A allele was found at a higher frequency in the control group (33.7%) compared to the AR group (26.6%). The GA genotype, however, was statistically significantly higher in the control group (53.5%) compared to the AR group (38%) (*p* < 0.05). When these data are evaluated, it is suggested that the GA genotype or the A allele itself may potentially confer protection against the AR. Nevertheless, genotypic findings were not associated with disease phenotype or biochemical findings. According to the data obtained from the GTEx portal, no results were found regarding the association between the rs3121310 polymorphism and *WNT3A* expression in whole blood, and there was no significant relationship in the lung, a respiratory-associated organ. Expression data for nasal mucosa were not available on the portal. Thus, the rs3121310 polymorphism does not appear to be associated with *WNT3A* expression. We suggest that other genetic and/or epigenetic factors modulate *WNT3A* expression in patients with AR. These negative results may be attributed to the relatively small cohort size. Therefore, our data should be validated through further studies with larger cohorts of patients and functional analyses are needed to test this hypothesis.

The anti-inflammatory and pro-inflammatory functions of the Wnt/β-catenin signaling pathway are known [8]. Consequently, many other studies have investigated the association of this pathway with allergic diseases. Reuter et al. reported that the Wnt/β-catenin signaling pathway could have a role in the regulation of dendritic cell-mediated allergic reactions within the lungs [19]. In a study conducted by Li et al., inhibition of the Wnt/β-catenin pathway in an OVA-induced mouse model of AR resulted in decreased numbers of eosinophils and mast cells. Additionally, downregulation of serum IgE, INF-γ, IL-1β, IL-4, IL-17, β-catenin, and GSK-3β was observed. Consequently, the study suggests that overexpression of the *RORA* gene or inactivation of the Wnt/β-catenin signaling pathway may alleviate nasal mucosal damage and eosinophil infiltration, reduce mast cell infiltration in nasal mucosal tissues, improve red blood cell immune adhesion, and mitigate AR. In the OVA-induced AR mouse model, inhibition of the Wnt/β-catenin signaling pathway and increased RORA activity are highlighted as potential therapeutic approaches for AR treatment [8]. Although various therapies have been developed for the treatment of AR, there are currently no data on therapeutic approaches associated with the Wnt/β-catenin signaling pathway. In a study conducted by Qiu et al. in 2021, the expression profiles of circRNA, miRNA, and mRNA in the nasal mucosa of adult AR patients were examined. The study reported that RNAs with altered expression profiles, including those involved in the Wnt pathway, play roles in significant biological processes [20]. In addition, there are other studies indicating that the expression profiles of various RNAs change in AR, and these RNAs are associated with the Wnt signaling pathway [21,22]. In another study, protein analysis conducted using two-dimensional gel electrophoresis and mass spectrometry on nasal lavage fluid of AR patients revealed an increase in the expression of Wnt-2b protein levels compared to controls [23]. Histamine production by mast cells causes symptoms such as nasal itching, nasal discharge, and sneezing in AR [24]. It has been demonstrated that the Histamine H1 receptor, activated by histamine, regulates gene expression through various signaling pathways, including the protein kinase C (PKC)/extracellular signal-regulated kinases (ERK) pathway, as well as the Wnt signaling pathway [25]. All of this evidence indicates that the Wnt signaling pathway is involved in the development and progression of AR. Our results are also consistent with these findings.

Asthma is associated with AR. Indeed, the clinical manifestation of AR can progress to asthma over time and both conditions can coexist together [26]. The role of the Wnt signaling pathway in the development and differentiation of airway cells is well established, and its association with asthma has been demonstrated both by in vivo and in vitro studies. Therefore, this pathway is also considered an important therapeutic target for asthma [27]. Inhibitors targeting the Wnt signaling pathway are being evaluated for the treatment of idiopathic pulmonary fibrosis. However, considering the diverse functions of the Wnt signaling pathway, caution is warranted when targeting this pathway, as it may have various effects on different cellular processes [28].

The complexity of Wnt signaling, characterized by numerous ligands, receptors, and co-receptors with their extensive interactions, poses challenges in deciphering the pathway’s cellular and organ-level functional implications, particularly in the context of infection and immune regulation [29]. While there is a common suggestion that WNT signaling is activated in inflammatory diseases, contrasting findings have also been reported [30,31]. Therefore, the inflammatory state does not necessarily imply upregulation of WNT ligands; additionally, there can be multiple reasons affecting the activation of this pathway. Especially considering human factors, the variety of environmental influences that can exert an effect is diverse, and data obtained from clinical studies can vary even across populations.

Although the difference was not statistically significant (*p* > 0.05), the serum WNT3A level was found to be higher in patients with mild AR (4.828 ± 4.054 ng/mL) compared to those with moderate/severe AR (3.109 ± 2.636 ng/mL). One possible reason for this could be the relatively small number of patients in each group, which may have limited the ability to perform a robust statistical analysis. It is difficult to explain the role of WNT3A in the progression, and stages of AR with our data. According to the ROC analysis, the impact of WNT3A in the differential diagnosis of AR was determined to be moderate (AUC: 0.67, 95% CI: 0.59–0.75). It is particularly noteworthy that an elevation in serum WNT3A protein levels is associated with an increased risk for AR. The current data represent the first clinical evidence supporting all the above-mentioned studies demonstrating the association of the Wnt pathway with AR and airway diseases. One limitation of our study is the relatively small size of our patient cohort. There are some limitations of this study: the first is the relatively small size of our patient cohort, which affects the generalizability of the findings; the other is the absence of functional analyses, which complicates the understanding of the biological implications of the observed genetic variations and biochemical findings. The study also suggests that the variability in environmental factors and genetic backgrounds may make it challenging to generalize the findings, and potential confounding factors may not have been accounted for.

## 5. Conclusions

The elevated serum WNT3A levels in AR patients align with studies suggesting that WNT3A plays a role in inflammation and immune modulation. The Wnt/β-catenin pathway, activated by WNT3A, has been linked to inflammatory processes in various diseases, including asthma and possibly AR. The higher frequency of the GA genotype in controls and the potential protective effect against AR suggest that the rs3121310 polymorphism might influence AR susceptibility. The lack of association with *WNT3A* expression in the available data could mean that the impact of this SNP on AR is indirect or involves other mechanisms. Further studies involving more AR patients will clarify whether WNT3A can serve as a biomarker for AR, and perhaps measurement of serum WNT3A protein levels will become routine in AR diagnosis. Risk profiling based on these biomarkers could facilitate early detection of complications in AR patients and implementation of preventive treatments. The data obtained from our project are important for elucidating the pathogenesis of AR and developing alternative diagnostic and therapeutic methods.

## Figures and Tables

**Table 1:** The oligonucleotide primers used for PCR, annealing temperature, and fragment sizes for genotyping.

SNP	Oligonucleotide Primer Sequences	Annealing Temperature (°C)	Fragment Sizes (bp)
rs752107 (C>T)	F: 5′-AGCAGGACTCCCACCTAAAC-3′ R: 5′-GCCTCATCCACCATAAAACC-3′	60.5	CC: 302 + 165 CT: 302 + 165 + 118 + 47 TT: 302 + 118 + 47
rs3121310 (G>A)	F: 5′-ATGCTCGGTGCCCTCTAAC-3′ R: 5′-GGCTTACTGACATGTGGTGC-3′	60.5	GG: 267 + 100 + 43 GA: 367 + 267 + 100 + 43 AA: 367 + 43

**Table 2:** Demographic characteristics and clinical profiles of patients with allergic rhinitis (AR) and healthy controls.

Characteristics		AR (n = 92)	Control (n = 86)	*p*
Male (%)/ Female (%)		32 (34.8%)/ 60 (65.2%)	21 (24.4%)/ 65 (75.6%)	0.1429 *
Mean age (years) ± SD		37.04 ± 12.47	33.16 ± 11.67	0.0550 **
Duration of AR diagnosis (months) (min–max)		28.46 (18–72)		
Severity of AR	Mild	64 (69.6%)		
	Moderate/Severe	28 (30.4%)		
Symptom duration	Intermittent	75 (81.5%)		
	Persistent	17 (18.5%)		

* Fisher’s exact test, ** Mann–Whitney test.

**Table 3:** Comparison of serum WNT3A levels according to patients’ disease characteristics.

	Symptom Duration			Severity of AR		
	Intermittent (n = 75)	Persistent (n = 17)	*p* *	Mild (n = 64)	Moderate/Severe (n = 28)	*p* *
WNT3A levels ng/mL (mean± SD)	4.133 ± 3.733	5.064 ± 3.851	0.0984	4.828 ± 4.054	3.109 ± 2.636	0.0532

* Mann–Whitney test, SD, standard deviation.

**Table 4:** Distributions of SNP genotypes and allele frequencies in patient and control groups.

SNP		AR n (%)	Control n (%)	OR ** (95% CI)	*p* *
rs752107	Genotype	n = 92	n = 86		
	CC	71 (77.2%)	71 (82.6%)	Ref	Ref
	CT	16 (17.4%)	8 (9.3%)	0.5 (0.2073–1.234)	0.184
	TT	5 (5.4%)	7 (8.1%)	1.4 (0.4574–4.075)	0.765
	CT + TT	21 (22.8%)	15(%)	0.6818 (0.3205–1.452)	0.356
	Allele	n = 184	n = 172		
	C	158 (85.9%)	150 (87.2%)	Ref	Ref
	T	26 (14.1%)	22 (12.8%)	0.8913 (0.486–1.616)	0.758
rs3121310	Genotype				
	GG	50 (54.4%)	34 (39.5%)	Ref	Ref
	GA	35 (38%)	46 (53.5%)	0.5174 (0.280–0.978)	**0.0433**
	AA	7 (7.6%)	6 (7%)	0.7933 (0.2607–2.731)	0.767
	GA + AA	42 (45.6%)	52 (60.5%)	1.821 1.008–3.371	0.0523
	Allele	n = 184	n = 172		
	G	135 (73.4%)	114 (66.3%)	Ref	Ref
	A	49 (26.6%)	58 (33.7%)	0.7134 (0.4482–1.125)	0.165

* Fisher’s exact test, ** OR, odds ratio, CI, confidence interval, Ref, reference, genotype comparisons between patient and control groups for rs752107 were calculated using 2 × 2 contingency tables as CC vs. CT, CC vs. TT, CC vs. CT + TT, and allele comparisons as C vs. T using 2 × 2 contingency tables. For rs3121310, genotype comparisons were calculated as GG vs. GA, GG vs. AA, GG vs. GA + AA using 2 × 2 contingency tables, and allele comparisons as G vs. A using 2 × 2 contingency tables. Bold value indicates statistical significance.

**Table 5:** The rare allele frequency in the patient and control groups and deviation from the Hardy–Weinberg equilibrium (HWE).

SNP	Rare Allele Frequency		*p* (HWE)	
	AR	Control	AR	Control
rs752107	0.141	0.128	**0.0147**	**0.00001**
rs3121310	0.266	0.337	0.7917	0.0929

Bold values indicate statistical significance.

**Table 6:** Comparison of patients’ clinical data based on their genotypes.

rs3121310	WNT3A Levels (ng/mL)		IgE Levels (UI/mL)		Symptom Duration				Severity of AR			
	Mean ± SD	*p* *	Mean ± SD	*p* *	Intermittent n (%)	Persistent n (%)	OR (95% CI)	*p* **	Mild n (%)	Moderate/Severe n (%)	OR (95% CI)	*p* **
GG	4.410 ± 3.975	Ref.	170.9 ± 147.5	Ref.	42 (45.6%)	8 (8.7%)	Ref.	Ref.	36 (39.1%)	14 (15.2%)	Ref.	Ref.
GA	4.157 ± 3.484	>0.999	172.8 ± 206.6	>0.999	26 (28.3%)	9 (9.8%)	1.817 (0.6203–5.663)	0.2858	23 (25%)	12 (13.1%)	1.342 (0.5269–3.331)	0.6341
AA	4.300 ± 3.927	>0.999	166.9 ± 137.1	>0.999	7 (7.6%)	0 (0%)	0.000 (0.000–2.857)	0.5769	5 (5.4%)	2 (2.2%)	1.003 (0.8223–1.389)	>0.999
GA + AA	4.181 ± 3.511	>0.999	171.9 ± 195.3	>0.999	33 (35.9%)	9 (9.8%)	1.432 (0.5025–4.335)	0.5936	28 (30.4%)	14 (15.3%)	1.286 (0.5509–2.971)	0.6520

* Kruskal-Wallis, Dunn’s multiple comparisons test, ** Fisher’s exact test, SD, standard deviation, CI, confidence interval, Ref, reference.

## Data Availability

The raw data supporting the conclusions of this article will be made available by the authors on request.
